# Prevalence and associated risk factors of stunting, wasting/thinness, and underweight among primary school children in Kandahar City, Afghanistan: a cross-sectional analytical study

**DOI:** 10.1186/s12889-024-19858-z

**Published:** 2024-08-27

**Authors:** Bilal Ahmad Rahimi, Aziz Ahmad Khalid, Wais Mohammad Lali, Wahid Ahmad Khalid, Javed Ahmad Rahimi, Walter R. Taylor

**Affiliations:** 1 Department of Pediatrics, Faculty of Medicine, Kandahar University, Afghan International Islamic University, Kabul, Afghanistan; 2 Department of Economics, Jamia Millia Islamia, Central University, New Delhi, India; 3 Research Center, Kandahar University, Kandahar, Afghanistan; 4 Department of Economics, Savitribai Phule Pune University, Pune, Maharashtra India; 5 Department of Business Administration, Gujarat University, Ahmedabad, Gujarat India; 6 Department of Medicine, Mahidol Oxford Tropical Medicine Clinical Research Unit (MORU), Mahidol University, Bangkok Thailand Centre for Tropical Medicine and Global Health, Nuffield, University of Oxford, Oxford, United Kingdom; 7 Department of Pediatrics, Faculty of Medicine, Kandahar University, Durahi, Beside Aino Mena Town, District 10, Kandahar, Afghanistan

**Keywords:** Stunting, Wasting, Thinness, Underweight, Malnutrition, Undernutrition, Afghanistan, Kandahar

## Abstract

**Background:**

Undernutrition, which includes stunting, wasting, and underweight, is a global problem, especially among children of low- and middle-income countries. To our knowledge, this study is first of its type from Afghanistan. Its main objectives were to estimate the prevalence and associated risk factors of stunting, wasting/thinness, and underweight among urban primary school children in Kandahar city of Afghanistan.

**Methods:**

This school-based cross-sectional study was conducted among 1205 primary school children aged 6–12 years during a period of six months (October 2022–March 2023). Anthropometric measurements and other data were collected from all the participants. Data were analyzed by using descriptive statistics, Chi square test (using crude odds ratio or COR), and multivariate logistic regression (using adjusted odds ratio or AOR).

**Results:**

Among the 1205 enrolled government school students, 47.4%, 19.5%, and 25.6% had stunting, wasting/thinness, and underweight, respectively. Statistically significant factors associated with stunting were age group 6–9 years (AOR 1.3, 95% CI 1.1–1.7), being girl (AOR 2.3, 95% CI 1.8–3.0), poverty (AOR 2.2, 95% CI 1.5–3.2), large family (AOR 3.0, 95% CI 2.4–3.9), illiterate mother (AOR 1.6, 95% CI 1.0–2.6), jobless head of the family (AOR 3.3, 95% CI 2.3–4.8), and skipping breakfasts (AOR 1.7, 95% CI 1.2–2.3). Main factor associated with wasting/thinness were age group 6–9 years (AOR 30.5, 95% CI 11.8–78.7), skipping breakfasts (AOR 22.9, 95% CI 13.9–37.8), and history of sickness during the past two weeks (AOR 17.0, 95% CI 6.6–43.8). Also, main factors associated with underweight were age group 6–9 years (AOR 2.6, 95% CI 1.6–4.1), skipping breakfasts (AOR 2.6, 95% CI 1.8–3.6), and poor sanitation (AOR 1.9, 95% CI 1.1–3.2).

**Conclusions:**

Stunting, wasting/thinness, and underweight are highly prevalent among primary school children (both girls and boys) in Kandahar city. It is recommended that local government (Afghanistan Ministry of Education and Ministry of Public Health) with the help of international organizations and donor agencies should implement comprehensive school-based feeding programs especially for girls. Health and nutrition education programs should be conducted with emphasis on nutrition of children aged 6–9 years as well as importance of healthy breakfast and good sanitation.

## Background

Malnutrition includes undernutrition, inadequate vitamins or minerals, overweight, obesity, and resulting diet-related noncommunicable diseases. Undernutrition consists of stunting (low height-for-age due to chronic or recurrent undernutrition), wasting (low weight-for-height due to recent and severe weight loss), and underweight (low weight-for-age) [1]. Globally in 2022, approximately 149 million and 45 million of the children under the age of five years were living with stunting and wasting, respectively. Approximately half of under five deaths are linked to undernutrition. Most of this mortality occurs in low- and middle-income countries (LMICs) [1].

Although globally many studies have been conducted on child undernutrition, majority of them have targeted the under-five children while school-aged have not been well addressed [2]. In 2021, a systematic review was conducted to study the nutritional status of school-age children and adolescents (5–19 years) reported in studies from LMICs around the world [3]. According to this study, highest prevalence of stunting, thinness, and underweight were reported from Democratic Republic of Congo (61.0% among 203 primary school children aged 7–17 years) [4], Ethiopia (58.3% among 211 adolescent girls with mean age of 14 years) [5], and Nigeria (95.7% among 139 primary school children aged 5–15 years) [6], respectively.

To the author’s knowledge, few published studies of undernutrition have been conducted in Afghanistan [7–9]. All of these studies are conducted among children under the age of five years. In 2014, a cross section community-based study was conducted in three of the 14 districts of Faryab province in northern Afghanistan. This study was conducted on 600 community children under-five years of age. Among these children, prevalence of wasting was 35.0%. This study revealed that statistically significant factors associated with acute malnutrition in under-five children were illiterate head of the household, household head with age ≤ 25 years, poor family, illiterate mother, child age < 24 months, history of diarrhea in the last two weeks, feeding frequency ≤ 3 times per 24 h, and having unprotected water source [9].

In 2013, a nutritional survey was conducted among under five (aged 0–59 months) children in the entire Afghanistan [8]. In this survey, the prevalence of moderate, severe, and overall stunting was 19.7%, 20.9% (95% CI 19.7–22.2), and 40.9% (95% CI 39.3–42.5), respectively. Meanwhile the prevalence of moderate, severe, and overall wasting was 5.5%, 4.0% (95% CI 3.5–4.6), and 9.5% (95% CI 8.7–10.4), respectively. Also, the prevalence of moderate, severe, and overall underweight was 15.2%, 9.7% (95% CI 8.9–10.6), and 25.0% (95% CI 23.8–26.3), respectively. According to 2013 Afghanistan national nutritional survey, the prevalence of stunting, wasting, and underweight among under five children in Kandahar were 42.2%, 13.5%, and 28.6%, respectively [8].

To the level of authors’ knowledge, there is not publish study from the entire Afghanistan that study the prevalence and associated risk factors of undernutrition among primary school children. This study is first of its type not only from Kandahar city, but from the entire Afghanistan. Main objectives of this school-based study were to estimate the prevalence and associated risk factors of stunting, wasting/thinness, and underweight among urban primary school children in Kandahar city of Afghanistan.

## Methods

### Study design and study area

This was a school-based cross-sectional analytical study, conducted during six-month-period (October 2022 – March 2023). Kandahar is Afghanistan's second largest city after Kabul, with a population of approximately 614,118 people. Kandahar city is located in the south-west of Afghanistan, at an elevation of 1,010 m above sea level. All the 145 schools of Kandahar city were selected for randomization using lottery method. After randomization, eight government schools (four boys’ schools and four girls’ schools) were selected for the study.

### Study population

Sample size was calculated using software of Epi Info version 7.2.2.6 (CDC, Atlanta, Georgia, USA). A 20% non-response rate was added. So, our sample size was 1278 children. Among these 1278 children, nine (0.7%) had chronic diseases (five had thalassemia, three had hemophilia, and one had type 1 diabetes mellitus), three (0.2%) had visible physical deformity (all three had paralytic poliomyelitis of lower limbs), and parents/care takers of 61 (4.8%) children refused to take part in the study. So, data was collected from 1205 children.

The study population was composed of school children with age 6–12 years and permanent residents of Kandahar. All the students in this study were living in urban area and no rural students were present in our study. All those children were excluded from the study who were having chronic diseases, had visible physical deformity, or refused (either child or guardian) to participate in the study.

### Ethical considerations

Ethical clearance was obtained from Ethics Committee of Kandahar University (code number KDRU-EC-2022.28) and permission from Kandahar Province Education Department authorities. Prior to the data collection, written informed consent from parents, and written informed assent from study participants were obtained with help of authorities. Interviews of participants using a predesigned questionnaire were conducted and anthropometric examinations were done as per standard operating procedures. For data collection, only patients' initials were used. Information of the participants will not be disclosed. Prior to entering into the computer for analysis, the collected data was coded and de-identified.

### Assessment of nutritional status in children

To assess the nutritional status of children, anthropometric measurements were collected. Body weight and height were obtained from child. All children stood barefooted against a vertical wall and height was obtained using a stadiometer to the nearest 0.1 cm. Body weight of children with lightweight clothes was measured to the nearest 0.1 kg using a digital balance which was validated before starting the measurement of weight. About 10% of the measurements were randomly selected for quality control and measured by another experienced researcher who was blinded for the previous measurement results. Also, to minimize the errors, data was double entered.

### Data analysis

The data were entered into Microsoft Excel 2019, cleaned, and imported to Statistical Package for the Social Sciences (SPSS) version 22 (Chicago, IL, USA) for statistical analysis. Descriptive analysis including frequency, mean, standard deviation (SD), and range was used to summarize demographic characteristics. Frequency and percentage were used to summarize categorical variables. Chi-square test (using crude odds ratio [COR]) was performed to assess the binary association between various categorical variables. All variables that were statistically significant in univariate analyses were assessed for independence in a multivariate logistic regression (using adjusted odds ratio [AOR]) to determine the factors associated with the predisposition of school children for getting stunting, wasting/thinness, or underweight. To control confounding bias, variables (such as age, sex, and economic status) were adjusted. A *P*-value of < 0.05 was considered statistically significant.

### Definitions

#### Malnutrition

The following anthropometric indices, height-for-age Z (HAZ) score, weight-for-age Z (WAZ) score, weight-for-height Z (WHZ) score, and BMI-for-age Z (BAZ) score were computed as per the WHO Child Growth Standards median (WHO, 2009) to assess the growth and nutritional status of the children [10].

- **Stunting:** (shortness), or low height-for-age, is a sign of chronic undernutrition. Children are defined as stunted if their HAZ score < –2 SD of WHO Child Growth Standards median.
- **Wasting/Thinness:**
  - In children 6–9 years of age, WHZ score was used to find wasting (defined as WHZ score < –2 SD).
  - In children 10–12 years of age, BAZ score was used to find thinness (defined as BAZ score < –2 SD).
- **Underweight:** or low weight-for-age, is a composite index that considers both acute and chronic undernutrition. Children are defined as underweight if their WAZ score is < –2 SD of the WHO Child Growth Standards median.
- **Overweight:** Children are defined as overweight if their BAZ score is >  + 1 SD.
- **Obesity:** Children are defined as obese if their BAZ score is >  + 2 SD.

#### Dietary diversity

It is defined as the number of various foods or food groups consumed over a given reference period [11]. In this study, dietary diversity was achieved (responded ‘yes’) when the student consumed at least three dietary groups (cereals, vegetables, and animal products) per day.

#### Food security

It was defined as a condition that all parents of the pupil access safe, sufficient, and nutritious food all times to meet their dietary needs [12].

#### Poverty

Family which earn < 170 Afghanis (< 1.90 USD) per person per day [13].

## Results

**Fig. 1:**
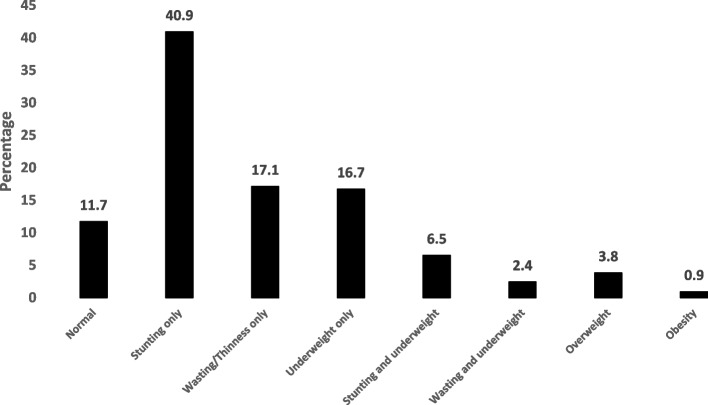
Nutritional status of school children (shown as percentage) in Kandahar, Afghanistan

Among the 1205 enrolled school students, 83.6% (1007/1205) had undernutrition, with 47.4% (571/1205), 19.5% (235/1205), and 25.6% (309/1205) having stunting, wasting/thinness, and underweight, respectively (Fig. 1). Most (880/1205 [73.0%]) of students belonged to poor families, with 997/1205 (82.7%) having illiterate mothers. Among these children, 71.3% had illiterate father, 33.2% were first born in birth order, 22.7% had history of sickness in the past two weeks, 84.7% did not have food security, 91.5% did not have dietary diversity, and 89.8% had poor sanitation. Among the study participants, number of boys (624/1205 [51.8%]) was slightly more than girls. Stunting, belonging to poor families, family size of ≥ 5 people, illiterate mothers, not washing hands after defecation/before eating, and habit of nail biting were more frequently observed among girls (Table 1).

**Table 1:** Nutritional, socio-demographic, and other characteristics of the school children

Variable	Frequency (%), *N* = 1205	Boys *N* = 624	Girls *N* = 581	*P*-value
**Nutritional status**				
Stunting only	493 (40.9)	232 (40.6)	339 (59.4)	< 0.001
Wasting or thinness only	206 (17.1)	121 (51.5)	114 (48.5)	0.921
Underweight only	201 (16.7)	164 (53.1)	145 (46.9)	0.599
Stunting and underweight	78 (6.5)	35 (44.9)	43 (55.1)	0.094
Wasting and underweight	29 (2.4)	16 (55.2)	13 (44.8)	0.085
Normal	141 (11.7)	74 (52.5)	67 (47.5)	0.786
Overweight	46 (3.8)	24 (52.2)	22 (47.8)	0.792
Obese	11 (0.9)	6 (54.5)	5 (45.5)	0.674
**Age (years)**				
6–9	443 (36.8)	217 (49.0)	226 (51.0)	0.138
10 – 12	762 (63.2)	407 (53.4)	355 (46.6)	
**Family economic status**^a^				0.005
Poor	880 (73.0)	434 (49.3)	446 (50.7)	
Not poor	325 (27.0)	190 (58.5)	135 (41.5)	
**Family size**				0.030
< 5 people	630 (52.3)	345 (54.8)	285 (45.2)	
≥ 5 people	575 (47.7)	279 (48.5)	296 (51.5)	
**Mother’s literacy**				< 0.001
Literate	208 (17.3)	132 (63.5)	76 (36.5)	
Illiterate	997 (82.7)	492 (49.3)	505 (50.7)	
**Father’s (guardian of family) occupation**				
On job	994 (82.5)	521 (52.4)	473 (47.6)	0.342
Jobless	211 (17.5)	103 (48.8)	108 (51.2)	
**Handwashing after defecating/before eating**				
Yes	939 (77.9)	509 (54.2)	430 (45.8)	0.002
No	266 (22.1)	115 (43.2)	151 (56.8)	
**Toilet availability**				
Yes	1108 (92.0)	582 (52.5)	526 (47.5)	0.081
No (open defecation)	97 (8.0)	42 (43.3)	55 (56.7)	
**Sanitation**				
Good	123 (10.2)	60 (48.8)	63 (51.2)	0.482
Poor	1082 (89.8)	564 (52.1)	518 (47.9)	
**Take breakfast before coming to school**				
Yes	1013 (84.1)	526 (51.9)	487 (48.1)	0.822
No	192 (15.9)	98 (51.0)	94 (49.0)	
**Habit of nail biting**				
Yes	186 (15.4)	76 (40.9)	110 (59.1)	0.001
No	1019 (84.6)	548 (53.8)	471 (46.2)	

^a^Family economic status: Poor = Family which earn < 170 Afghanis (< 1.90 USD) per person per day. Not poor = Family which earn ≥ 170 Afghanis (≥ 1.90 USD) per person per day

There was no statistically significant difference between stunted and normal children in father literacy (*p*-value 0.803), birth order (*p*-value 0.242), history of sickness in last two weeks (*p*-value 0.822), food security (*p*-value 0.725), and dietary diversity (*p*-value 0.284). Among students with stunting, logistic regression revealed that factors associated with stunting were age 6–9 years (AOR 1.3, 95% CI 1.1–1.7, and *p*-value 0.014), female gender (AOR 2.3, 95% CI 1.8–3.0, and *p*-value < 0.001), belonging to poor family (AOR 2.2, 95% CI 1.5–3.2, and *p*-value < 0.001), family size ≥ 5 people (AOR 3.0, 95% CI 2.4–3.9, and *p*-value < 0.001), illiterate mother (AOR 1.6, 95% CI 1.0–2.6, and *p*-value 0.038), jobless father or guardian of the family (AOR 3.3, 95% CI 2.3–4.8, and *p*-value < 0.001), and not taking breakfast before coming to school (AOR 1.7, 95% CI 1.2–2.3, and *p*-value 0.001) (Table 2).

**Table 2:** Univariate analyses and logistic regression of the factors associated with stunting among school children

Variable	Total, N (%), *N* = 1205	Stunting		COR (95% CI)	*P*-value	AOR (95% CI)^a^	*P*-value
		**Present, N (%), ***N*** = 571**	**Absent, N (%), ***N*** = 634**				
**Age**							
6–9 years 10 – 12 years	443 (36.8) 762 (63.2)	231 (52.1) 340 (44.6)	212 (47.9) 422 (55.4)	1.4 (1.1–1.7) 1	0.012	1 1.3 (1.1–1.7)	0.014
**Gender**							
Male Female	624 (51.8) 581 (48.2)	232 (37.2) 339 (58.3)	392 (62.8) 242 (41.7)	1 2.4 (1.9–3.0)	< 0.001	1 2.3 (1.8–3.0)	< 0.001
**Family economic status**^b^							
Poor	880 (73.0)	479 (54.4)	401 (45.6)	3.0	< 0.001	1	< 0.001
Not poor	325 (27.0)	92 (28.3)	233 (71.7)	(2.3–4.0) 1		2.2 (1.5–3.2)	
**Family size**							
< 5 people ≥ 5 people	630 (52.3) 575 (47.7)	208 (33.0) 363 (63.1)	422 (67.0) 212 (36.9)	1 3.5 (2.7–4.4)	< 0.001	1 3.0 (2.4–3.9)	< 0.001
**Mother’s literacy**							
Literate Illiterate	208 (17.3) 997 (82.7)	51 (24.5) 520 (52.2)	157 (75.5) 477 (47.8)	1 3.4 (2.4–4.7)	< 0.001	1 1.6 (1.0–2.6)	0.038
**Father’s (guardian of family) occupation**							
With job Jobless	994 (82.5) 211 (17.5)	414 (41.6) 157 (74.4)	580 (58.4) 54 (25.6)	1 4.1 (2.9–5.7)	< 0.001	1 3.3 (2.3–4.8)	< 0.001
**Habit of nail biting**							
Yes No	186 (15.4) 1019 (84.6)	100 (53.8) 471 (46.2)	86 (46.2) 548 (53.8)	1.4 (1.0–1.9) 1	0.058	–	–
**Handwashing after defecating/before eating**							
Yes No	939 (77.9) 266 (22.1)	436 (46.4) 135 (50.8)	503 (53.6) 131 (49.2)	1 1.2 (0.9–1.6)	0.213	–	–
**Toilet availability**							
Yes	1108	520 (46.9)	588 (53.1)	1	0.286	–	
No (open defecation)	(92.0) 97 (8.0)	51 (52.6)	46 (47.4)	0.8 (0.5–1.2)			–
**Sanitation**							
Good	123 (10.2)	54 (43.9)	69 (56.1)	1	0.414	–	
Poor	1082 (89.8)	517 (47.8)	565 (52.2)	1.2 (0.8–1.7)			–
**Take breakfast before coming to school**							
Yes	1013 (84.1)	459 (45.3)	554 (54.7)	1	0.001	1	0.001
No	192 (15.9)	112 (58.3)	80 (41.7)	1.7 (1.2–2.3)		1.7 (1.2–2.3)	

*AOR* Adjusted Odds Ratio, *CI* Confidence interval, *COR* Crude odds ratio, *N* Number

^a^Variables with non-significance on bivariate analysis were not included in logistic regression model

^b^Family economic status: Poor = Family which earn < 170 Afghanis (< 1.90 USD) per person per day. Not poor = Family which earn ≥ 170 Afghanis (≥ 1.90 USD) per person per day

There was no statistically significant difference between wasted/thin and normal children in mother literacy (*p*-value 0.763), father occupation (*p*-value 0.723), habit of nail biting (*p*-value 0.289), toilet availability (*p*-value 0.410), and sanitation (*p*-value 0.813). Among students with wasting or thinness, logistic regression showed that factors associated with wasting or thinness were age 6–9 years (AOR 30.5, 95% CI 11.8–78.7, and *p*-value < 0.001), not taking breakfast before coming to school (AOR 22.9, 95% CI 13.9–37.8, and *p*-value < 0.001), and had sickness in the past two weeks (AOR 17.0, 95% CI 6.6–43.8, and *p*-value < 0.001) (Table 3).

**Table 3:** Univariate analyses and logistic regression of the factors associated with wasting/thinness among school children

Variable	Total, N (%), *N* = 1205	Wasting or Thinness		COR (95% CI)	*P*-value	AOR (95% CI)^a^	*P*-value
		**Present, N (%), *N* = 235**	**Absent, N (%), *N* = 970**				
**Age**					< 0.001		< 0.001
6–9 years	443 (36.8)	159 (35.9)	284 (64.1)	5.1		1	
10 – 12 years	762 (63.2)	76 (10.0)	686 (90.0)	(3.7–6.9) 1		30.5 (11.8–78.7)	
**Gender**							
Male	624 (51.8)	121 (19.4)	503 (80.6)	1	0.920	–	
Female	581 (48.2)	114 (19.6)	467 (80.4)	1.0 (0.8–1.4)			–
**Family economic status**^b^							
Poor	880 (73.0)	181 (20.6)	699 (79.4)	1.3 (0.9–1.8)	0.124	–	
Not poor	325 (27.0)	54 (16.6)	271 (83.4)	1			–
**Family size**							
< 5 people	630 (52.3)	69 (11.0)	561 (89.0)	1	< 0.001	1	0.029
≥ 5 people	575 (47.7)	166 (28.9)	409 (71.1)	3.3 (2.4–4.5)		0.4 (0.1–0.9)	
**Father’s literacy**							
Literate	346 (28.7)	131 (37.9)	215 (62.1)	4.4 (3.3–6.0)	< 0.001	1	0.724
Illiterate	859 (71.3)	104 (12.1)	755 (87.9)	1		1.2 (0.5–2.9)	
**Birth order**							
First born	400 (33.2)	57 (14.2)	343 (85.8)	1	0.001	1	0.308
Later born	805 (66.8)	178 (22.1)	627 (77.9)	1.7 (1.2–2.4)		1.3 (0.8–1.9)	
**Handwashing after defecating/before eating**							
Yes	939 (77.9)	198 (21.1)	741 (78.9)	1	0.009	–	
No	266 (22.1)	37 (13.9)	229 (86.1)	0.6 (0.4–0.9)			–
**Take breakfast before coming to school**							
Yes	1013 (84.1)	137 (13.5)	876 (86.5)	1	< 0.001	1	< 0.001
No	192 (15.9)	98 (51.0)	94 (49.0)	6.7 (4.8–9.3)		22.9 (13.9–37.8)	
**Sickness in the past two weeks**							
Yes	273 (22.7)	123 (45.1)	150 (54.9)	6.0 (4.4–8.2)	< 0.001	1	< 0.001
No	932 (77.3)	112 (12.0)	820 (88.0)	1		17.0 (6.6–43.8)	
**Food security present**							
Yes	184 (15.3)	15 (8.2)	169 (91.8)	1	< 0.001		
No	1021 (84.7)	220 (21.5)	801 (78.5)	0.3 (0.2–0.6)		–	–
**Dietary diversity present**							
Yes	103 (8.5)	5 (4.9)	98 (95.1)	1	< 0.001	–	
No	1102 (91.5)	230 (20.9)	872 (79.1)	0.2 (0.1–0.5)			–

*AOR* Adjusted Odds Ratio, *CI* Confidence interval, *COR* Crude odds ratio, *N* Number

^a^Variables with non-significance on bivariate analysis were not included in logistic regression model

^b^Family economic status: Poor = Family which earn < 170 Afghanis (< 1.90 USD) per person per day. Not poor = Family which earn ≥ 170 Afghanis (≥ 1.90 USD) per person per day

There was no statistically significant different between underweight and normal children in father literacy (*p*-value 0.853), father occupation (*p*-value 0.616), birth order (*p*-value 0.057), habit of nail biting (*p*-value 0.221), and history of sickness in last two weeks (*p*-value 0.528). Among students with underweight, based on logistic regression, factors associated with underweight were age 6–9 years (AOR 2.6, 95% CI 1.6–4.1, and *p*-value < 0.001), not taking breakfast before coming to school (AOR 2.6, 95% CI 1.8–3.6, and *p*-value < 0.001), and having poor sanitation (AOR 1.9, 95% CI 1.1–3.2, and *p*-value 0.022) (Table 4).

**Table 4:** Univariate analyses and logistic regression of the factors associated with underweight among school children

Variable	Total, N (%), *N* = 1205	Underweight		COR (95% CI)	*P*-value	AOR (95% CI)^a^	*P*-value
		**Present, N (%), *N* = 309**	**Absent, N (%), *N* = 896**				
**Age**					< 0.001		< 0.001
6–9 years	443 (36.8)	170 (38.4)	273 (61.6)	2.8 (2.1–3.6)		1	
10 – 12 years	762 (63.2)	139 (18.2)	623 (81.8)	1		2.6 (1.6–4.1)	
**Gender**							
Male	624 (51.8)	164 (26.3)	460 (73.7)	1	0.599	–	
Female	581 (48.2)	145 (25.0)	436 (75.0)	0.9 (0.7–1.2)			–
**Family economic status**^b^							
Poor	880 (73.0)	213 (24.2)	667 (75.8)	0.8 (0.6–1.0)	0.063	–	
Not poor	325 (27.0)	96 (29.5)	229 (70.5)	1			–
**Family size**							
< 5 people	630 (52.3)	112 (17.8)	518 (82.2)	1	< 0.001	1	0.751
≥ 5 people	575 (47.7)	197 (34.3)	378 (65.7)	2.4 (1.8–3.1)		1.1 (0.7–1.7)	
**Mother’s literacy**							
Literate	208 (17.3)	66 (31.7)	142 (68.3)	1	0.027	–	
Illiterate	997 (82.7)	243 (24.4)	754 (75.6)	0.7 (0.5–1.0)			–
**Take breakfast before coming to school**							
Yes	1013 (84.1)	228 (22.5)	785 (77.5)	1	< 0.001	1	< 0.001
No	192 (15.9)	81 (42.2)	111 (57.8)	2.5 (1.8–3.5)		2.6 (1.8–3.6)	
**Toilet availability**							
Yes	1108 (92.0)	292 (26.4)	816 (73.6)	1.7 (1.0–2.9)	0.056	–	
No (open defecation)	97 (8.0)	17 (17.5)	80 (82.5)	1			–
**Handwashing after defecating/before eating**							
Yes	939 (77.9)	253 (26.9)	686 (73.1)	0.7 (0.5–1.0)	0.052	–	
No	266 (22.1)	56 (21.1)	210 (78.9)	1			–
**Sanitation**							
Good	123 (10.2)	18 (14.6)	105 (85.4)	1	0.003	1	0.022
Poor	1082 (89.8)	291 (26.9)	791 (73.1)	2.1 (1.3–3.6)		1.9 (1.1–3.2)	
**Food security present**							
Yes	184 (15.3)	16 (8.7)	168 (91.3)	1	< 0.001	–	
No	1021 (84.7)	293 (28.7)	728 (71.3)	0.2 (0.1–0.4)			–
**Dietary diversity present**							
Yes	103 (8.5)	7 (6.8)	96 (93.2)	1	< 0.001	–	
No	1102 (91.5)	302 (27.4)	800 (72.6)	0.2 (0.1–0.4)			–

*AOR* Adjusted Odds Ratio, *CI* Confidence interval, *COR* Crude odds ratio, *N* number

^a^Variables with non-significance on bivariate analysis were not included in logistic regression model

^b^Family economic status: Poor = Family which earn < 170 Afghanis (< 1.90 USD) per person per day. Not poor = Family which earn ≥ 170 Afghanis (≥ 1.90 USD) per person per day

## Discussion

The prevalence of stunting among primary school children in this study was 47.4%. Nearly same prevalence of stunting has been observed in Nigeria (prevalence was 46.2% among rural school children and 33.8% among urban school children) [14]. Compared to this study, higher prevalence of stunting has been reported from Democratic Republic of Congo (prevalence was 61.0% among 197 primary school children aged 7–17 years) [4], Ethiopia (prevalence was 57% among 633 rural community 6–12 years old school age children) [15], and Ghana (prevalence was 50.3% among 650 school children) [16]. However, lower prevalence of stunting has been reported from Sudan (prevalence was 22.1% among 2638 school children aged 5–15 years) [17], India (prevalence was 36.0% among 100 rural school children aged 6–14 years) [18], and Sri Lanka (prevalence was 30.3% among 4484 school students aged 5–10 years) [19]. Similarly lower prevalence was reported from Kenya (prevalence was 30.2% among 384 school children aged 6–12 years living in a low-income urban community) [20], China (prevalence was 11.7% among 1474 rural school children aged 5–12 years) [21], and India (prevalence was 18.5% among urban school age children) [22]. In addition, lower prevalence of stunting was observed in Pakistan (prevalence was 8.2% among 1860 urban primary school children aged 5–12 years) [23] and Sudan (prevalence was 7.1% among 835 primary school children aged 6–14 years) [24]. These differences of prevalence observed during different countries (or different areas of the same country) could be due to differences in sample size, socioeconomic status, living condition, health policy, child feeding practices, school feeding programs, and child health care [25].

The prevalence of wasting/thinness in this study was 21.2%. Nearly same prevalence of wasting/thinness was observed in Ghana (prevalence was 19.4% among 650 school children) [16], Sri Lanka (prevalence was 20.9% among 4484 school students aged 5–10 years) [19] and Sudan (prevalence was 23.1% among 835 primary school children aged 6–14 years) [24]. Contrary to this study, increased prevalence of wasting/thinness was reported from India (prevalence was 33.3% among urban school age children) [22], Sudan (prevalence was 32.3% among 2638 school children aged 5–15 years) [17], and Democratic Republic of Congo (prevalence was 29.7% among 197 primary school children aged 7–17 years) [4]. Lower prevalence of wasting/thinness than this study were observed in Pakistan (prevalence was 10.1% among 1860 urban primary school children aged 5–12 years) [23], Ethiopia (prevalence was 9.8% among 396 urban primary school children aged 5–12 years) [26], Kenya (prevalence was 4.5% among 384 school children aged 6–12 years living in a low-income urban community) [20], and India (prevalence was 2.0% among 100 rural school children aged 6–14 years) [18]. The relatively increased prevalence of wasting/thinness in this study might be attributed to the decreased availability of safe water supplies in the city and the decreased prevalence of hand washing that might in turn increase the risk of infection and undernutrition [2].

The prevalence of underweight in this study was 28.7%. Nearly same prevalence has been reported from Democratic Republic of Congo (prevalence was 26.0% among 197 primary school children aged 7–17 years) [4] and Sri Lanka (prevalence was 25.9% among 4484 school students aged 5–10 years) [19]. However, compared to this study, higher prevalence of underweight were reported from Nigeria (prevalence was 95.7% among 139 primary school children aged 5–15 years) [6], Myanmar (prevalence was 44% in boys while 29% in girls among 558 school-aged children between 5–10 years) [27], and India (prevalence was 38.4% among urban school age children) [22]. Meanwhile, compared to this study, lower prevalence of underweight has been reported from Kenya (prevalence was 14.9% among 384 school children aged 6–12 years living in a low-income urban community) [20], India (prevalence was 9.0% among rural school children aged 6–14 years) [18], Ethiopia (prevalence was 5.7% among 396 urban primary school children aged 5–12 years) [26], South Africa (prevalence was 5.4% among 167 school children aged 6–12 years) [28], and Cameroon (prevalence was 0.6% among 822 primary school children aged 5–17 years) [29]. Difference in the prevalence of undernutrition in different countries (or even different parts of the same country) of the world could be due to the difference in the socio-economic status, culture, health education, parents’ literacy level, sample size, definition of stunting, eating habits of the children, distance of school from home, and presence/absence of school feeding programs.

Factors associated with stunting among primary school children in this study were age 6–9 years, female gender, belonging to poor family, family size ≥ 5 people, illiterate mother, jobless father or guardian of the family, and not taking breakfast before coming to school. A study conducted among 1860 urban primary school children aged 5–12 years in Pakistan reported that factors associated with stunting were age > 8 years (AOR 3.6, 95% CI 1.9–6.9), urban area with low socio-economic status (AOR 2.6, 95% CI 1.2–5.8), and low-income neighborhoods (AOR 4.6, 95% CI 1.6–13.1) [23]. An Ethiopian study on 551 school age children reported that factors significantly associated with stunting were being male caregiver (AOR 4.26, 95% CI 1.3–14.5), family size ≥ 4 (AOR 4.7, 95% CI 1.9–11.7), and separated kitchen room (AOR 0.1, 95% CI 0.02–0.5) [2]. A study conducted in Sudan among 2638 school children aged 5–15 years revealed that main factors associated with stunting were age group 13–15 years (OR 3.8, 95% CI 2.8–5.1), being boys (OR 1.4, 95% CI 1.1–1.7), and children from rural areas (OR 2.4, 95% CI 1.8–3.3) [17].

In this study, factors associated with wasting/thinness among primary school children were age 6–9 years, not taking breakfast before coming to school, and presence of sickness in the past two weeks. A study conducted among 1860 urban primary school children aged 5–12 years in Pakistan reported that factor associated with thinness was urban area with low socio-economic status (AOR 2.3, 95% CI 1.2–4.3) [23]. An Ethiopian study on 551 school age children reported that factors significantly associated with thinning were drinking coffee (AOR 2.3, 95% CI 2.0–5.2) and child dietary diversity score < 4 (AOR 2.5, 95% CI 1.7–8.9) [2]. A study conducted in Sudan among 2638 school children aged 5–15 years showed that main factors associated with thinness in school children were being boy, rural residence, child whose families depend on unsafe source of drinking water, children who skipped meal during the school day, and children who bring their food from their houses [17].

In this study, factors associated with underweight among primary school children were age 6–9 years, not taking breakfast before coming to school, and having poor sanitation. A study conducted in Ethiopia among 396 urban primary school children aged 5–12 years showed that child age 6–8 years (AOR 12.9, 95% CI 2.4–71.2) and 3–4 children in the family (AOR 8.2, 95% CI 1.3–50.7) were the key determinants for underweight [26]. Another study conduct4ed in southern Ethiopia among 450 school children aged 4–14 concluded that maternal education status (AOR 0.3, 95% CI 0.1–0.9) and household food insecurity (AOR 3.9, 95% CI 1.2–12.0) were independently associated with underweight among school children [30]. A study conducted in Palestine among 1320 school children aged 6–12 years reported that statistically significant factor associated with underweight was being female (OR 23.5, 95% CI 3.9–141.8) [31]. Risk factor of having large family could be due to the reason that the per-capita calories consumption are less in large families [18]. The developmental, economic, social, and medical impacts of the global burden of malnutrition are serious and lasting, i.e., for people, their families, communities, and even countries [1]. One of the main reasons of increased prevalence of undernutrition in Kandahar could be due to increased burden of communicable diseases among children in Kandahar province [32–35] and vice versa.

### Limitations

Despite being a useful study, there were limitations in our study. First, the micronutrient status of children was not measured, and its real association with stunting and wasting was not known. Second, as this was a cross-sectional study, no causal link can be inferred. Third, this research only used a structured interviewer-administered questionnaire and measurements that might not reflect culture and perception of food taboos in the community. Fourth, this research was confined to only one city (Kandahar) of Afghanistan. This affects the generalizability of the findings to the whole country or other cities and provinces of Afghanistan. Fifth, information about variables related to time were answered from memory. Therefore, recall bias cannot be ruled out.

## Conclusions

Finding of this study showed that stunting, wasting/thinness, and underweight are highly prevalent among primary school children in Kandahar city. Main factors associated with stunting were age group 6–9 years, being girl, poverty, large family, illiterate mother, jobless father or guardian of the family, and skipping breakfast. Main factor associated with wasting or thinness were age group 6–9 years, skipping breakfast, and sickness during the past two weeks. Main factors associated with underweight were age group 6–9 years, skipping breakfast, and poor sanitation.

It is recommended that local government (Afghanistan Ministry of Education and Ministry of Public Health) with the help of international organizations and donor agencies (such as WFP, WHO, UNICEF, and USAID) conduct health and nutrition education programs for the improvement of health and nutritional status of school children, especially girls, in Kandahar. We also recommend that awareness should be created among the school age children, parents and teachers, on the dietary requirements of both boys and girls, with emphasis on nutrition of children aged 6–9 years and the importance of healthy breakfast. Government should make better plans and policies to decrease unemployment and poverty as well as provide good sanitation to the people. Special educational programs should be implemented to educated illiterate mothers in Kandahar. Furthermore, comprehensive school-based feeding programs are required to be implemented to alleviate under nutrition among school age children in Kandahar city. Further nutritional studies are needed to be conducted among the rural school children of Kandahar province. Also, large-scale national nutritional survey is of utmost importance to be conducted among the school children of all 34 provinces of Afghanistan.

## Data Availability

All the data and materials related to this study are available on request.

## Abbreviations

AOR: Adjusted odds ratio
BAZ: BMI-for-age Z score
BMI: Body mass index
CI: Confidence interval
COR: Crude odds ratio
HAZ: Height-for-age Z score
SD: Standard deviation
SPSS: Statistical Package for the Social Sciences
UNICEF: United Nations Children's Fund
USAID: United States Agency for International Development
USD: United States Dollar
WAZ: Weight-for-age Z score
WFP: World Food Program
WHO: World Health Organization
WHZ: Weight-for-height Z score

## Data availability

All the data and materials related to this study are available on request to the corresponding author.
